# Comparing intermittent and daily prednisone in duchenne muscular dystrophy: a systematic review and meta-analysis

**DOI:** 10.1097/MS9.0000000000003049

**Published:** 2025-02-27

**Authors:** Eeshal Zulfiqar, Sonia Hurjkaliani, Shahood Ahmed Umar, Maryam Shahzad, Muneeba Ahsan, Quareeha Tahir, Urooj Nizami, Bariha Rizvi, Abdullah Abid Khan, Aniqa Baloch, Syed Shafaat Hussain, Mehwish Rabbani, Marhaba Fatima, Muhammad Omar Larik, Muhammad Hasanain, Muhammad Umair Anjum, Pratik Bhattarai

**Affiliations:** a Department of Medicine, Dow Medical College, Karachi, Pakistan; b Department of Medicine, Ziauddin Medical College, Karachi, Pakistan; c Department of Medicine, Karachi Medical and Dental College, Karachi, Pakistan; d Department of Medicine, Fazaia Ruth Pfau Medical College, Karachi, Pakistan; e Department of Medicine, Bahria University of Health Sciences, Karachi, Pakistan; f Department of Medicine, Shaheed Mohtarma Benazir Bhutto Medical College, Karachi, Pakistan; g Department of Medicine, People’s University of Medical and Health Sciences for Women, Nawabshah, Pakistan; h Department of Medicine, Dow International Medical College, Karachi, Pakistan; i Department of Medicine, Manipal College of Medical Sciences, Pokhara, Nepal

**Keywords:** daily prednisone, DMD, duchenne muscular dystrophy, intermittent prednisone, meta-analysis

## Abstract

**Background:**

Duchenne muscular dystrophy (DMD) is an X-linked disorder caused by DMD gene mutations, leading to muscle wasting due to dystrophin deficiency. Current treatment with corticosteroids like prednisone shows benefits but lacks clarity on optimal dosing regimens. This systematic review and meta-analysis aim to determine the efficacy and safety of daily versus intermittent prednisone dosing in DMD management.

**Methods:**

We conducted a systematic search of PubMed, Google Scholar, Embase, and Scopus databases to identify studies comparing daily versus intermittent prednisone in DMD treatment. The study protocol was registered with PROSPERO (CRD42024549050).

**Results:**

After the systematic search, 6 trials were included in the pooled analysis. Intermittent prednisone was associated with a higher prevalence of cushingoid appearance (RR: 1.72; 95% CI: 1.17 to 2.51; *P* = 0.005), excessive hair growth (RR: 1.56; 95% CI: 1.08 to 2.24; *P* = 0.02), and hypertension (RR: 3.42; 95% CI: 1.87 to 6.25; *P* < 0.0001). In contrast, there were no statistically significant differences between daily versus intermittent prednisone in terms of forced vital capacity (FVC), blood pressure, loss of ambulation, weight changes, weight gain, bone fracture, behavioral changes, and DEXA lumbar spine Z-scores.

**Conclusion:**

No significant differences in efficacy outcomes regimens were observed. However, intermittent prednisone was associated with a higher prevalence of certain adverse effects, such as cushingoid appearance, excessive hair growth, and hypertension. These findings provide valuable insights for clinicians when choosing treatment strategies and highlight the need for personalized approaches to minimize side effects while maintaining efficacy.

**HIGHLIGHTS**

- Prednisone has shown benefit in DMD, but optimized dosing regimen remain unknown.
- In this meta-analysis, we pooled 6 studies comparing daily vs intermittent prednisone.
- No particular differences in efficacy outcomes were observed.
- Cushingoid appearance, hair growth, and hypertension was increased in intermittent prednisone.
- Further large-scale trials are necessary to validate our conclusion.

## Introduction

Duchenne muscular dystrophy (DMD) is a progressive muscle-wasting condition resulting from mutations in the DMD gene, which hinder dystrophin production^[1]^. The absence of dystrophin causes gradual muscle loss, replaced by connective tissue and fat, leading to a decline in function^[2]^. DMD is an x-linked recessive disorder, more prevalent in males, with less than 10 cases per 10 000 males^[1]^. Typically, boys are diagnosed with this condition around the age of five, characterized by gait abnormalities, difficulty getting up from the floor, and an inability to run. Without intervention, the progression of contractures and muscle weakness can result in the inability to walk between the ages of 7 and 13^[3]^.

The current management of the disease involves a comprehensive approach that includes rehabilitation, the initiation of steroids, addressing side effects, and providing psychosocial support. While drugs targeting the underlying mutation and exon skipping are currently in development, there is no definitive cure for this disease. However, the use of corticosteroids has proven to be effective in reducing inflammation and improving survival rates in affected patients^[4]^. Although the exact mechanism of corticosteroids in Duchenne muscular dystrophy is not understood, their potential beneficial effects include inhibition of muscle proteolysis, stimulation of myoblast proliferation, stabilization of muscle fiber membranes, and differential regulation of genes in muscle fibers^[3]^. Long-term glucocorticoid therapy has been shown to delay the loss of milestones and reduce the risk of death over the course of the study^[5]^. Nevertheless, both corticosteroid regimens have their own set of side effects, including weight gain, behavioral changes, and growth delays. The primary corticosteroids used in the management of DMD are prednisone and deflazacort (a synthetic analog of prednisone)^[4]^. Prednisone can be administered at a daily dose of 0.75 mg/kg/day for 6 months or 10 mg/kg/weekend over 12 months^[6]^.

In light of conflicting and sparse results observed in published literature regarding the comparison of daily vs. intermittent prednisone, a first-ever systematic review and meta-analysis was conducted to generate a robust conclusion regarding the preferred strategy in terms of their efficacy and safety profile. While alternative methodologies, such as scientometric analysis, could provide insights into publication trends, they are not designed to synthesize clinical evidence or evaluate comparative outcomes. Therefore, a systematic review and meta-analysis were the most appropriate approach to address our clinical research question. Ultimately, we aim to create a deeper understanding of these two regimens, in order to promote evidence-based recommendations for clinicians and patients alike.

## Methods

This qualitative and quantitative synthesis followed the Preferred Reporting Items for Systematic Reviews and Meta-Analyses (PRISMA) guidelines^[7]^, ensuring a comprehensive and unbiased analysis of available evidence. Additionally, the study protocol was registered with the PROSPERO International Prospective Register of Systematic Reviews (CRD42024549050).

### Data sources and search strategy

We performed a thorough electronic search of multiple databases, including PubMed, Google Scholar, Embase, and Scopus from inception to June 2024. The aim was to identify studies comparing daily versus intermittent prednisone administration. Our search strategy had predefined Medical Subject Headings (MeSH) terms and free text keywords such as “Duchenne muscular dystrophy,” or “prednisone,” or “daily,” or “intermittent,” combined with Boolean operators “AND” and “OR” to increase accuracy. The detailed search strategy is available in Supplementary Table 1 (available at: http://links.lww.com/MS9/A739).

### Eligibility criteria

Inclusion criteria: All studies reporting primary data on patients diagnosed with DMD undergoing either daily prednisone or intermittent prednisone treatment were eligible for further screening and potential inclusion. Subsequently, studies reporting the following outcomes were included: efficacy outcomes; FVC %, FVC liters, systolic BP, diastolic BP, loss of ambulation, weight change, body mass index (BMI), and safety outcomes; weight gain, bone fractures, behavioral change, cushingoid appearance, excessive hair growth, hypertension, and DEXA lumbar spine Z-scores. In addition, eligible study designs included randomized controlled trials (RCTs) and prospective or retrospective observational cohort studies.

Exclusion criteria: All other study types (letters, reviews, case reports, case series) were excluded. Additionally, we excluded studies that did not report the outcomes of interest or were not published in the English language.

### Data extraction and quality assessment

Data from the selected studies were extracted independently by two reviewers (MA and QT). Any discrepancies at any stage were resolved by a third reviewer (MS). Extracted data included study characteristics, sample size, duration of treatment, and reported outcomes. Quality assessment of the observational studies was conducted using the Newcastle-Ottawa Scale^[8]^, while of the clinical trials by the modified Cochrane Risk of Bias Tool (version 2.0), specifically designed for RCTs^[9]^.

### Statistical analysis

The data was synthesized using Cochrane Review Manager software (RevMan version 5.4.1). A random-effects model was applied, utilizing risk ratio (RR) and mean difference (MD) as the effect measures, with 95% confidence intervals (CIs) and statistical significance determined at *P* <0.05 for pooling the results of individual studies. The heterogeneity among the studies was assessed using Higgin’s I^2^ test^[10]^, with I^2^ values interpreted as follows: 0-25% indicating low heterogeneity, 25-75% indicating moderate heterogeneity, and >75% indicating high heterogeneity.

## Results

### Literature search, characteristics of studies and risk of bias assessment

**Figure 1.**
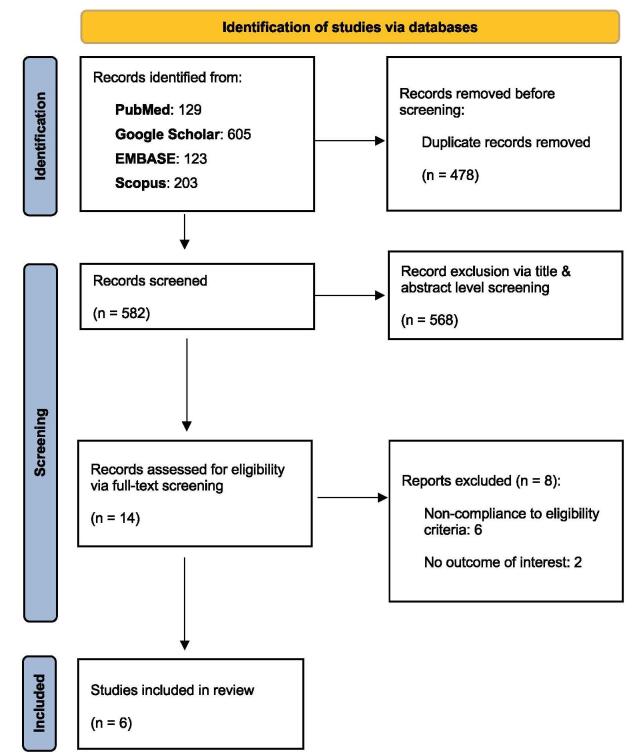
PRISMA flowchart showcasing the systematic search process.

A total of 1,060 initial studies were retrieved. After the removal of duplicates and full-texts screening, 6 studies^[11-16]^ were shortlisted for inclusion in this systematic review and meta-analysis, resulting in the pooling of 708 patients. The detailed literature search process can be summarized in the PRISMA flowchart, illustrated in Fig. 1. The baseline clinical characteristics of the included study population is available in Table 1.

**Table 1:** Study and baseline patient characteristics of the included population

Study	Year	Country	Study design	Participants, n		Dosage (mg/kg)		Mean age, years (SD)		Mean weight, kilograms (SD)		Duration (months)
				Intervention	Control	Intervention	Control	Intervention	Control	Intervention	Control	
Escolar, *et al*	2011	Florida, United States	RCT	32	32	5	0.75	5.8 (0.9)	5.7 (0.7)	22.4(5.3)	24.4(8.4)	12
Guglieri, *et al*	2022	Canada, Germany, United Kingdom Italy, United States	RCT	66	65	0.75	0.75	5.9 (1.1)	5.8^[1]^	20.2 (3.5)	20^[3]^	36
Fenichel, *et al*	1991	NR	RCT	31	33	1.25	0.75	NR	NR	25.56 (0.15)	25.56 (0.15)	6
Ricotti, *et al*	2012	United Kingdom	Prospective observational study	136	154	0.6	0.5	NR	NR	NR	NR	> 48
Goto, *et al*	2016	Japan	Retrospective observational study	51	36	1	0.75	9.5 (2.8)	12.4 (2.8)	NR	NR	>6
Gurpreet, *et al*	2022	India	RCT	36	36	0.75	0.75	7.4 (1.1)	7.3 (1.7)	17.9 (2.9)	18 (2.9)	NR

RCT: randomized controlled trial; n: number of patients, SD: standard deviation; NR: not reported.

Out of the 6 included studies, 4 were RCTs and were assessed by Cochrane’s RoB-2 tool, which evaluates domains such as randomization process, deviations from intended interventions, missing outcome data, measurement of outcomes, and selection of reported results. Three studies reported a low^[11-13]^ risk of bias, and one study had some concerns^[16]^. Two of the included studies were observational studies and were evaluated using the Newcastle-Ottawa Scale^[14,15]^, which assesses the quality across three domains: selection of study groups, comparability of groups, and ascertainment of outcomes. Ricotti 2012 scored 7 out of 9, with 2 points deducted due to the lack of a well-defined non-exposed cohort and limited adjustment for potential confounding, indicating moderate quality. Conversely, Goto 2016 achieved a score of 9 out of 9 reflecting high quality. Further information with regards to the evaluation of RCTs is available in Supplementary Figure 1 (available at: http://links.lww.com/MS9/A739), and judgements with regards to observational studies are available in Supplementary Table 2 (available at: http://links.lww.com/MS9/A739).

### Efficacy outcomes

**Figure 2.**
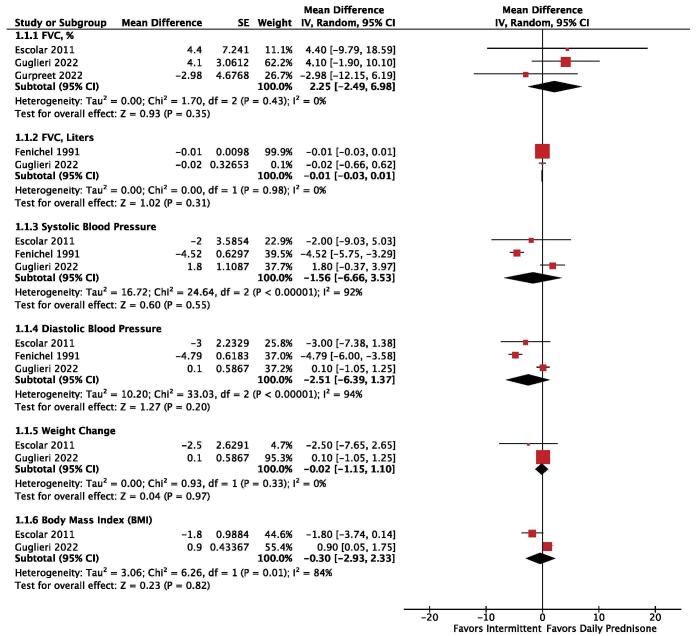
Forest plot showcasing efficacy outcomes of daily versus intermittent prednisone in DMD.

**Figure 3.**
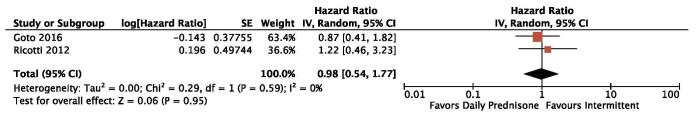
Forest plot showcasing loss of ambulation in daily versus intermittent prednisone in DMD.

The following efficacy outcomes were investigated: (i) FVC, %, (ii) FVC, liters, (iii) systolic blood pressure, (iv) diastolic blood pressure, (v) weight change, and (vi) BMI. Three studies (n = 267) reported FVC (%), revealing the lack of statistical differences between daily and intermittent prednisone (MD: 2.25; 95% CI: −2.49 to 6.98; *P* = 0.35; Fig. 2). Two studies (n = 195) reported FVC (liters), revealing the lack of significant differences between the two treatment regimens (MD: −0.01; 95% CI: −0.03 to 0.01; *P* = 0.31; Fig. 2). Systolic blood pressure was assessed in three studies (n = 259), revealing no statistical differences between the two treatment regimens, with high heterogeneity (MD: −1.56; 95% CI: −6.66 to 3.53; *P* = 0.55; I^2^: 92%; Fig. 2). Diastolic blood pressure, reported by three studies (n = 259), also revealed no significant differences between the two treatment regimens, exhibiting high heterogeneity (MD: −2.51, 95% CI: −6.39 to 1.37; *P* = 0.20; I^2^: 94%; Fig. 2). Two studies (n = 195) reported data on weight change, revealing the lack of statistical differences between daily and intermittent prednisone (MD: −0.02; 95% CI: −1.15 to 1.10; *P* = 0.97; Fig. 2). BMI, reported by two studies (n = 195), revealed no statistical differences between daily and intermittent prednisone, with significant heterogeneity (MD: −0.30; 95% CI: −2.93 to 2.33; *P* = 0.82; I^2^: 84%; Fig. 2). Two studies (n = 377) reported loss of ambulation, revealing the lack of statistical differences between daily and intermittent prednisone (HR: 0.98; 95% CI: 0.54 to 1.77; *P* = 0.95; Fig. 3). In summary, there were no significant differences between daily and intermittent prednisone in terms of efficacy outcomes investigated.

### Safety outcomes

**Figure 4.**
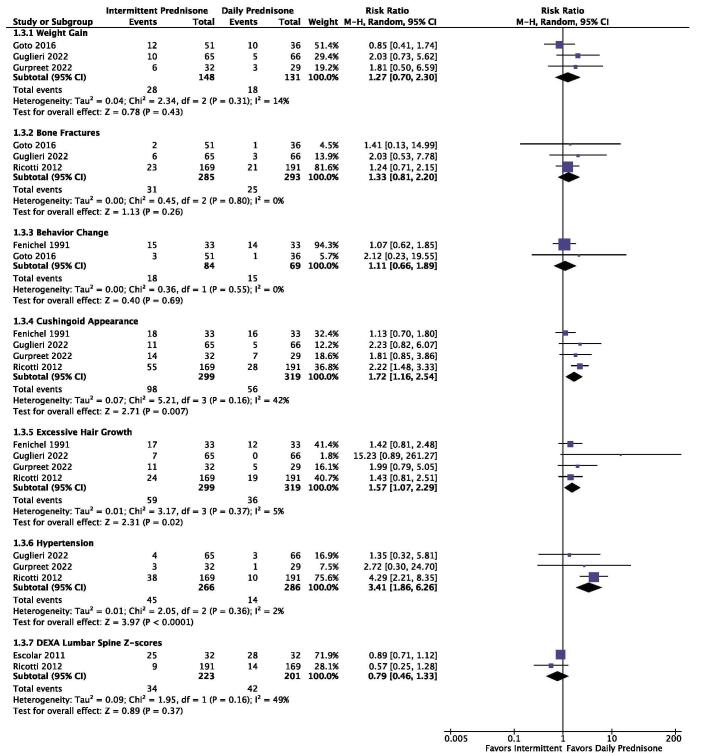
Forest plot showcasing safety outcomes of daily versus intermittent prednisone in DMD.

In this meta-analysis, the following adverse effects were observed: (i) weight gain, (ii) bone fracture, (iii) behavior changes, (iv) cushingoid appearance, (v) excessive hair growth, (vi) hypertension, and (vii) DEXA lumbar spine Z-score. Three studies (n = 279) reported data on weight gain, revealing the lack of significance difference between daily and intermittent prednisone (RR: 1.27; 95% CI: 0.07 to 2.30; *P* = 0.43; I^2^: 14%; Fig. 4). Three studies (n = 578) reported on bone fractures, revealing a lack of significant difference between daily and intermittent prednisone (RR: 1.33; 95% CI: 0.81 to 2.20; *P* = 0.26; Fig. 4). Two studies (n = 153) reported on behavior changes, revealing a lack of significant difference between daily and intermittent prednisone (RR: 1.11; 95% CI: 0.66 to 1.89; *P* = 0.69, Fig. 4). Four studies (n = 618) compared the risk of developing cushingoid appearance, revealing a significant difference as the group administered intermittent prednisone had a higher prevalence of cushingoid appearance (RR: 1.72; 95% CI: 1.16 to 2.54; *P* = 0.0007; Fig. 4). Four studies (n = 618) reported on excessive hair growth, revealing a significant difference as the group administered intermittent prednisone had higher incidence of excessive hair growth compared to the group administered daily prednisone (RR: 1.57; 95% CI: 1.07 to 2.29; *P* = 0.02; Fig. 4). Three studies (n = 552) reported on hypertension, revealing a statistically significant difference as the group administered intermittent prednisone had a higher incidence of hypertension compared to the other group (RR: 3.41; 95% CI: 1.86 to 6.26; *P* < 0.0001; Fig. 4). Two studies (n = 424) reported data on DEXA Lumbar spine Z-score, to assess the effect on bone health. Analysis revealed no significant difference between the group given daily prednisone and the one given intermittent prednisone (RR: 0.79, 95% CI: 0.46 to 1.33; *P* = 0.37; Fig. 4).

## Discussion

Our systematic review and meta-analysis, encompassing a broad range of studies, investigates the use of daily versus intermittent prednisone in the treatment of DMD. The analysis revealed several key findings. In terms of efficacy outcomes, there were no statistically significant differences in any outcomes, including FVC, blood pressure, weight change, BMI, and loss of ambulation. However, in terms of safety outcomes, patients given intermittent prednisone were associated with a higher prevalence of cushingoid appearance, excessive hair growth, and hypertension. No noteworthy differences were identified with other safety outcomes.

DMD is a condition that only affects men, with an estimated incidence of 1 in 3800–6200 live male births^[17-19]^. The eventual loss of independent ambulation and the gradual involvement of upper limb function, which usually occur between the ages of 9 and 14 years, are characteristics of DMD^[14,20-22]^. The standard of therapy for delaying the progression of DMD disease involves pharmacologic treatment with the corticosteroids deflazacort or prednisone/prednisolone (henceforth referred to as “prednisone”)^[23,24]^. Prednisone has been used extensively to treat DMD for decades despite not having a Food and Drug Administration (FDA)-approved prescription for the condition^[25]^.

Treatment with corticosteroids has been demonstrated to improve respiratory function by strengthening muscles and stabilizing the spine^[26,27]^. According to our analysis, FVC% and FVC (liters) showed no heterogeneity and both treatment approaches maintain comparable pulmonary function in patients. These findings imply that clinicians can expect similar efficacy in preserving lung function irrespective of the dosing regimen chosen. Since height affects the anticipated respiratory value, the patients’ shorter stature may have had an impact on %FVC. Nevertheless, the intermittent group’s outcome cannot be explained by this impact. Research on DMD patients’ respiratory function has made use of a statistical technique that was modified for a longitudinal study design^[28]^. The mixed model can better account for imbalanced and unevenly spaced %FVC observations across time, and it can also evaluate age-related variations in %FVC with greater accuracy. Similarly, no significant differences were found in weight change or BMI between the two regimens. Stable weight gain outcomes across regimens suggest that other factors, such as patient preference and side effect profiles, can guide the choice of regimen without compromising on weight management. Gaining weight is the most frequent side effect of corticosteroid therapy in DMD patients. This increases the mechanical strain on the muscles that are deteriorating and probably leads to the termination of ambulation^[29]^.

It is important to note that significant variability is shown by the high heterogeneity (I^2^: 94%) in the diastolic blood pressure response across trials, indicating the need for more research. Variations in patient characteristics (age, gender, severity of disease, comorbid conditions), study design (sample size, duration of follow-up, measurement techniques), variability in the type, dosage, and adherence of interventions, statistical methods, geographic and environmental factors, and genetic variability may all be contributing factors to this variation. As a result, our findings should be interpreted with caution, particularly when generalizing to diverse patient populations or treatment scenarios. Determining these origins can improve our comprehension of blood pressure control in individuals with DMD.

No significant differences in the incidence of bone fractures were observed between the regimens suggesting that either regimen can be used without increased risk of compromising long-term bone health in DMD patients. Despite having normal birth weights and lengths, individuals with DMD experience delayed growth during the first few years of life, and by the age of ten, their median height is marginally below the 50th percentile^[30]^. The median height by the age of eighteen falls below the fifth percentile. Children with neuromuscular problems frequently experience osteoporosis and are more likely to suffer pathologic fractures^[29]^. Previous research suggests that prednisone treatment-induced increases in muscle strength and exercise may stabilize bone density^[29]^. To fully evaluate the impact of corticosteroid-use on bone metabolism and fracture risk, however, a lengthier investigation would be necessary. Intermittent prednisone was associated with a higher prevalence of cushingoid appearance (*P* = 0.005; I^2^: 39%), highlighting the need for clinicians to consider cosmetic and psychological implications when choosing a regimen. In a previous trial, a cross-over design was used to evaluate an intermittent regimen of prednisone (0.75 mg/kg/day given for the first 10 days of the month, for six months) with placebo. During the prednisone treatment phase, four subjects were observed to have a cushingoid look, compared to just one during the placebo period^[31]^. Similarly, other adverse effects like excessive hair growth, hypertension are also shown to be more common with Intermittent prednisone.

The findings from this meta-analysis provide valuable insights for clinical practice, supporting the use of either daily or intermittent prednisone based on individual patient needs and side effect profiles. This flexibility in treatment options can help optimize outcomes and patient adherence. Thus, it is essential to effectively manage adverse effects, especially those that are more common when using intermittent dosage. To reduce side effects, clinicians should closely monitor their patients and modify their treatment as necessary. Modifications in dosage, additional drugs to offset adverse effects, and routine follow-ups to monitor patient improvement are some strategies that may be employed.

It is important to acknowledge the limitations of our meta-analysis. Firstly, the high heterogeneity reported in some of the efficacy outcomes (systolic BP: 92%, diastolic BP: 94%, BMI: 84%) could reduce the reliability of results and have a potential for misleading conclusions. Secondly, potential biases in the included studies could threaten the validity of the results and the strength of the available evidence. Thirdly, a smaller number of available studies means less availability of data, and the overall strength of the evidence from this meta may be weaker. Fourthly, disease severity, as assessed by the 6-minute walk test and 10-meter walk test, was inconsistently reported across studies. While one study provided 6-minute walk test and 10-meter walk test data, others did not include comparable data, creating heterogeneity among the analysis owing to the varying/unknown disease severity. As a result, precautions are warranted when interpreting the efficacy of treatment. Lastly, data on concurrent therapies were not reported in the included studies, leaving an important potential confounding factor unaddressed.

The results of this meta-analysis identify numerous topics for future research. Future studies should focus on long-term results to determine the efficacy and safety of these therapy regimens over time. Furthermore, comparing established techniques to newer medications, such as gene therapy and other developing pharmacological treatments, may provide insights toward improving patient care. Investigating the differences in reactions among different patient subgroups may also improve tailored treatment techniques. Moreover, there is a need to hold further randomized clinical trials to confirm and validate the findings of these studies. Continuous trials also allow the collection of long-term data on the effectiveness and safety of therapies, which leads to a better understanding of their impact. Exploration of combination therapies with different drugs is a useful way to lead to better outcomes, ultimately improving clinical outcomes for people with DMD.

## Conclusion

The findings of this meta-analysis show that while there is no significant difference in the efficacy outcomes between the two dosing regimens, the safety outcomes indicate a higher risk of cushingoid appearance, excessive hair growth, and hypertension in those administered intermittent prednisone. However, variability in efficacy outcome, including high heterogeneity in systolic blood pressure, diastolic blood pressure, and BMI, highlights the challenges of synthesizing data from diverse study designs and populations. In addition, inconsistencies in reporting key confounding factors, such as disease severity and concurrent therapies, present significant challenges. Hence, the results of this meta-analysis should be interpreted with caution. Future research, comparing intermittent vs. daily prednisone in DMD, should aim for comprehensive datasets in order to establish a valid conclusion.

Considering DMD is a rare disease, it should be identified immediately, and prompt management should be undertaken to prevent its worsening. Effective management techniques, such as prednisone, physical therapy, and new gene therapies, seek to delay disease progression and improve functional outcomes, so studies should be conducted in these domains to find ways to effectively treat DMD. However, these advances must be evaluated against issues of long-term safety, convenience, and cost-effectiveness to prevent any kind of patient discomfort.

## Supplementary Material

## Data Availability

All data generated has been either published in the manuscript, or available in the supplementary file.

## Research registration unique identifying number (UIN)

Prospective registration via PROSPERO was completed for this systematic review and meta-analysis (UIN: CRD42024549050).
