# Supplementary material for: Comparing intermittent and daily prednisone in duchenne muscular dystrophy: a systematic review and meta-analysis
Source: Ann Med Surg (Lond). 2025 Feb 27;87(3):1637–45. doi: 10.1097/MS9.0000000000003049 (PMC11981458; doi:10.1097/MS9.0000000000003049)
Supplement: Supplementary file 1 [file ms9-87-1637-s001.docx]

| **No.** | **Database** | **Search Strategy** | **No. of articles** |
| --- | --- | --- | --- |
| **1** | PubMed | ("muscular dystrophy, duchenne"[MeSH Terms] OR ("muscular"[All Fields] AND "dystrophy"[All Fields] AND "duchenne"[All Fields]) OR "duchenne muscular dystrophy"[All Fields] OR ("duchenne"[All Fields] AND "muscular"[All Fields] AND "dystrophy"[All Fields])) AND "DMD"[All Fields] AND ("prednison"[All Fields] OR "prednisone"[MeSH Terms] OR "prednisone"[All Fields]) | 129 |
| **2** | Embase | ('Duchenne Muscular Dystrophy'/exp OR 'DMD') AND ('prednisone'/exp OR 'corticosteroids'/exp OR 'Adrenal Cortex Hormones'/exp) AND ('daily regimen' OR 'intermittent regimen' OR 'drug administration schedule'/exp OR 'intermittent drug therapy') | 123 |
| **3** | Google  Scholar | ("Duchenne Muscular Dystrophy") OR ("DMD") AND ("Prednisone") AND ("Daily") AND ("Intermittent") AND ("Regimen") | 605 |
| **4** | Scopus | TITLE-ABS-KEY ("Duchenne Muscular Dystrophy" OR "DMD") AND TITLE-ABS-KEY("prednisone" OR "corticosteroids" OR "Adrenal Cortex Hormones") AND TITLE-ABS-KEY("daily regimen" OR "intermittent regimen" OR "drug administration schedule" OR "intermittent drug therapy") | 203 |

**Supplementary Table 1.** Comprehensive list of search strategies employed for each database included within the systematic review.

| **Studies** | **S1** | **S2** | **S3** | **S4** | **C1** | **O1** | **O2** | **O3** | **Total** |
| --- | --- | --- | --- | --- | --- | --- | --- | --- | --- |
| **Ricotti 2012** | **★** |  | **★** | **★** | **★** | **★** | **★** | **★** | **7/9** |
| **Goto 2016** | **★** | **★** | **★** | **★** | **★★** | **★** | **★** | **★** | **9/9** |

**Supplementary Table 2**. Author judgements for risk of bias assessment of included observational studies using the Newcastle-Ottawa tool.


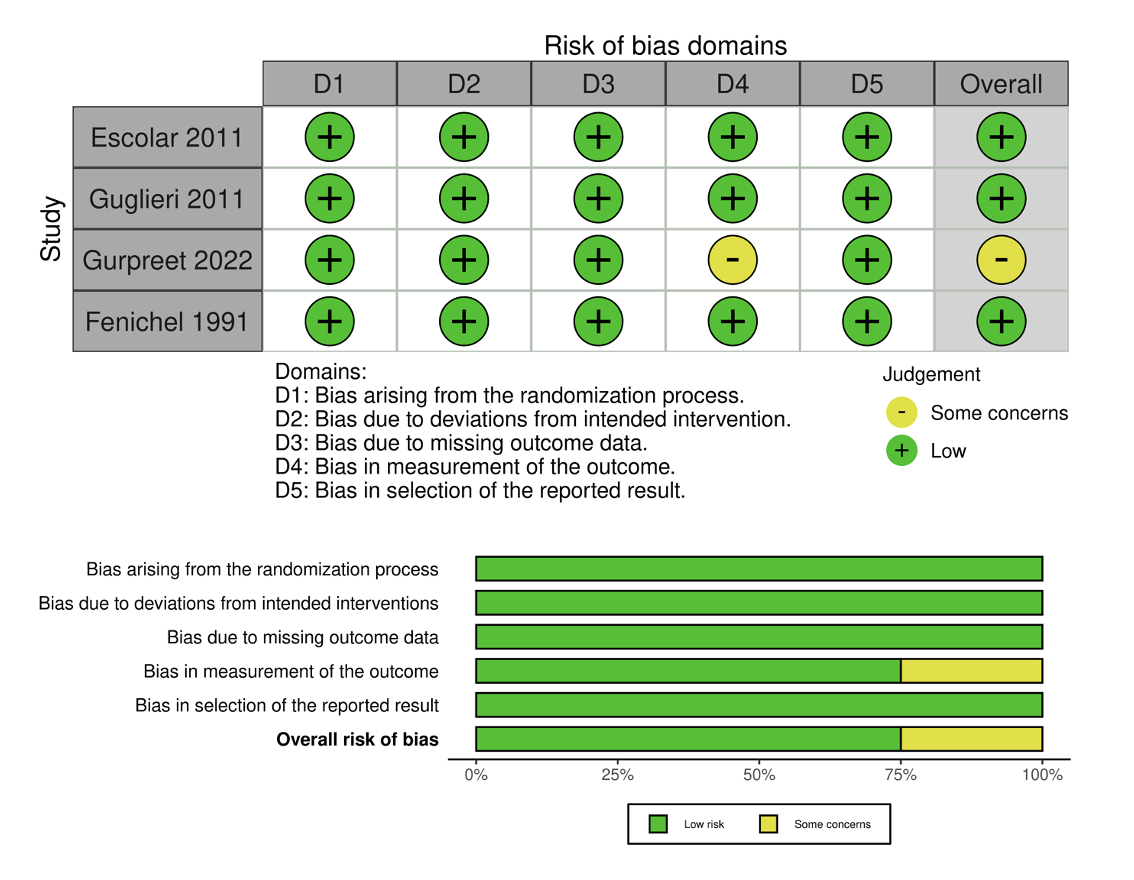


**Supplementary Figure 1.** Author judgements for risk of bias assessment of included randomized clinical trials (RCTs) using the Cochrane Risk of Bias Tool (version 2.0).
